# Significant difference of serum 25-hydroxyvitamin D level in male hemodialysis patients with or without diabetes; a single center study

**Published:** 2012-07-01

**Authors:** Hamid Nasri, Mahmoud Rafieian-Kopaei

**Affiliations:** ^1^Department of Internal Medicine, Shahrekord University of Medical Sciences, Iran; ^2^Medical Plants Research Center, Shahrekord University of Medical Sciences, Shahrekord, Iran

**Keywords:** Hemodialysis, Vitamin D, Diabetes

## Abstract

It is well found that the assessment of circulating 25-hydroxyvitamin D offers better information about vitamin D status in patients. This cross-sectional study was aimed to better understand the probable difference of 25-hydroxyvitamin D level in a group of hemodialysis (HD) patients with or without diabetes. 25-hydroxyvitamin D level (normal range of values is 25 to 125 nmol/L) was measured following an overnight fasting. The study was conducted on 36 subjects (15 female, 21 male), consisting of 25 (female=11, male=14) non-diabetic HD patients and 11 (female=4, male=7) diabetic HD patients. A significant difference of serum 25-hydroxyvitamin D level between diabetic and non-diabetic male HD patients with more values of 25-hydroxyvitamin D level in none-diabetic HD patients was found (r=0.014). Least studied was conducted on the influence of diabetes on 25-hydroxyvitamin D level in HD patients, lower vitamin D level in diabetes patients may aggravates their condition. Thus, further investigations need to define this aspect of hemodialysis patients.

**Implication for health policy/practice/research/medical education:**

In a study on 36 stable hemodialysis patients, we found a low serum 25-hydroxyvitamin D in diabetic male patients in comparison to non-diabetic males. Further investigations need to define this aspect of hemodialysis patients.

## Introduction

25-hydroxyvitamin D is the main circulating metabolite of vitamin D (1). It is well found that the assessment of circulating 25-hydroxyvitamin D offers better information vitamin D status in patients, while biological active form of vitamin D is 1,25(OH)2 vitamin D, synthesized in the kidney. Recently, various investigations have highlighted the role of plasma 25-hydroxyvitamin D level in mineral metabolism dysregulation in chronic renal failure (1,2). It has been established that a moderate reduction in plasma 25-hydroxyvitamin D level plays a role in the development of secondary form of hyperparathyroidism in patients under hemodialysis, while a greater reduction in plasma 25-hydroxyvitamin D level is associated with the risk of getting osteoporosis (1–3). Moreover, less attention has been made to 25-hydroxyvitamin D in hemodialysis patients with or without diabetes. This cross-sectional study was aimed to better understand the probable difference of 25-hydroxyvitamin D level in a group of hemodialysis patients with or without diabetes.

## Patients and Methods

### Patients

This cross-sectional study was carried out on a group of stable hemodialysis patients. According to the intensity of secondary hyperparathyroidism, each patient received oral active vitamin D_3_ (Calcitriol; Rocaltrol), calcium carbonate or Rena-Gel (Sevelamer) tablets at various doses.

The study was done in the hemodialysis (HD) section of Hajar Medical Educational and Therapeutic Center of Shahrekord University of Medical Sciences in Shahrekord, Iran.

### Laboratory methods

25-hydroxyvitamin D level (normal range of values is 25 to 125 nmol/L) was measured following an overnight fasting, by enzyme-linked immunosorbent assay (ELISA) method with DRG kits from Germany. Also serum pre-dialysis and post dialysis blood urea nitrogen (BUN) was measured. For the efficacy of HD, the urea reduction rate (URR) was calculated from pre- and post-blood urea nitrogen (BUN) data. Duration and proportion of hemodialysis were assessed from the patients’ records. The duration of each HD session was 4 hours.

### Ethical issues

The research followed the tenets of the declaration of Helsinki; written informed consent was obtained and the research was approved by ethical committee of Shahrekord University of Medical Sciences.

### Statistical analysis

Data were expressed as the mean±SD and median values. The comparison between the groups was done using the student’s t-test. The statistical analyses were performed using SPSS 11.5 (SPSS Inc., Chicago, USA)). The statistical significance was determined at a p<0.05.

## Results

The study was conducted on 36 (female 15, male 21), consisting of 25 (F=11, M=14) non-diabetic HD patients and 11 (F=4, M=7) diabetic HD patients. Table 1 summarizes some data of the patients. The mean patients’ age was 47±17 years. The mean value of serum 25-hydroxyvitamin D level of patients was 10.5±18.7 nmol/L. There were no significant differences of serum 25-hydroxyvitamin D level between male and female subjects or diabetic and non-diabetic patients (*p*; N.S). A significant difference of serum 25-hydroxyvitamin D level between diabetic and non-diabetic subjects of male HD patients with more values of 25-hydroxyvitamin D level in none-diabetic HD patients was found (r=0.014).

**Table 1:** Data of hemodialysis patients

**Total patients N=36**	**Mean ±SD**	**Median**
Age (years)	47±17	43
DH* (months)	32±36	19
Dialysis proportion	123±54	156
URR (%)	59±9	57.5
25-OH vit. D (nmol/l)	10.5±18.7	3.5

*Duration of hemodialysis

## Discussion

In this study we found a significant difference of serum 25-hydroxyvitamin D level between diabetic and non-diabetic subjects of male HD patients with more values of 25-hydroxyvitamin D level in none-diabetic HD patients was found. Gonzalez *et al*. conducted a study on 103 patients undergoing HD and found that 97% of the patients had vitamin D levels in the suboptimal range (4). In a study on 230 patients, Wang *et al*. found that a lower serum 25-hydroxyvitamin D level was associated with an increased risk of cardiovascular events in routine peritoneal dialysis patients (5). Limited studies have conducted on the impact of diabetes on 25-hydroxyvitamin D level in HD patients. Lower vitamin D level in diabetic patients may aggravate their conditions.

## Conclusion

In summary, we concluded that our findings are of interest for two reasons: first, because there is limited previous data concerning low serum 25-hydroxyvitamin D in diabetics in comparison to non-diabetic patients. However further investigations are needed to define this aspect of hemodialysis patients.

## Authors’ Contributions

HN and MRK wrote the manuscript equally.

## Conflict of interests

The authors declared no competing interests.

## Ethical considerations

Ethical issues (including plagiarism, data fabrication, double publication) have been completely considered by the authors.

## Funding/Support

None.
